# Intraocular pressure fluctuation following intravitreal dexamethasone implant and incidence of secondary ocular hypertension: a Zambian perspective

**DOI:** 10.11604/pamj.2021.39.108.23528

**Published:** 2021-06-06

**Authors:** Pallavi Raj, Kshitiz Kumar, Santosh Balasubramaniam, Coimbatore Sekar Geetha, Amar Agarwal

**Affiliations:** 1 Sankara Nethralaya, Mukundapur, Kolkata, India,; 2 Disha Eye Hospital, Barrackpore, Kolkata, India,; 3 Dr. Agarwal's Eye Hospital, Stand 599, Protea Road, Lusaka, Zambia,; 4 Dr. Agarwal's Eye Hospital, Alwarpet, Chennai, India

**Keywords:** Dexamethasone implant, ocular hypertension, intraocular pressure, Zambia eyes

## Abstract

**Introduction:**

to evaluate the effect of dexamethasone biodegradable implant (DEX-I), on intraocular pressure (IOP), to determine the incidence of secondary ocular hypertension (OHT) and to analyze the IOP changes as per the treatment indication in Zambian cohort.

**Methods:**

retrospective consecutive case series of patients receiving one DEX-I between January 2016 and September 2018 with a minimum follow-up of four months in a tertiary care centre in Zambia. The IOP was recorded before the injection and at 1^st^ week, 1^st^, 2^nd^, 3^rd^ and 4^th^ month after the injection. Ocular hypertension was defined as IOP ≥ 21 mmHg or an increase of ≥ 10 mmHg from baseline.

**Results:**

the effects of 122 injections given to ninety - nine patients (65 male: 65%; mean age 57.3) were included. The main indications for treatment were diabetic macular edema (DME, 52%), retinal vein occlusion (18%), post-surgical macular edema (18%) and non-infectious posterior uveitis (10%). Mean IOP before the injection was was 14.7mmHg and at 1^st^ week, 1^st^, 2^nd^, 3^rd^ and 4^th^ months after the injection it was 14.4 (p=0.08), 16.1 (p=0.01), 17.5 (p<0.001), 15.7 (p=0.006) and 14.9 (p=0.06) mmHg, respectively. The incidence of secondary OHT was 30.32% in this cohort. Peak incidence of OHT was between 1 - 2 months, with majority of cases in DME group (75%) and 43% diabetic eyes followed by 23% non-infectious posterior uveitis cases developing OHT post injection. OHT was well managed with anti-glaucoma medications only.

**Conclusion:**

DEX-I showed a good pressure tolerance in this cohort. Secondary ocular hypertension developed in one-third of patients receiving injection which was transient and successfully managed with topical anti-glaucoma medications only. Diabetic eyes are more prone to develop ocular hypertension and therefore needs close monitoring following injection.

## Introduction

The role of intravitreal dexamethasone implant (Ozurdex; Allergan, Irvine, CA) is well established as the first line of therapy in the management of macular edema following retinal vein occlusions, non-infectious posterior uveitis, diabetic macular edema (DME) and postsurgical macular edema [1-4]. However, like any other intravitreal steroid, a frequent concern with dexamethasone implant is development of secondary ocular hypertension (OHT) besides cataract formation [5].

The precise mechanism of IOP elevation after steroid treatment is not very clear, but is primarily attributed to reduced facility of aqueous outflow secondary to increased trabecular meshwork resistance, increased responsiveness of trabecular meshwork to glucocorticoids, decreased levels of prostaglandins, mechanical obstruction caused by crystalline steroid particles in case of triamcinolone acetonide injection and or due to genetic influence involving expression of MYOC gene at locus GLC1A along with several other postulated genes [6-11].

Unlike other intravitreal corticosteroids (triamcinolone acetonide and fluocinolone acetonide), dexamethasone implant seems to be better tolerated. The pivotal clinical studies, HURON and GENEVA, show a low rate of OHT after treatment with dexamethasone implant [2,12]. More recent studies have shown incidence of OHT ranging from 23% - 28.5% [13-16]. SAFODEX study also highlighted, patients with younger age, retinal vein occlusion, insulin dependent diabetes mellitus (IDDM), non-infectious posterior uveitis to have higher propensity for development of OHT [15]. Role of dexamethasone implant position within posterior chamber in relation to IOP spikes have been entailed in few studies [16].

There has been no study from Zambia or African continent which looks at the effect of dexamethasone implant on intraocular pressure. The purpose of this study was to analyse the pressure tolerance of dexamethasone implant (DEX-I, Ozurdex), to report the incidence of secondary ocular hypertension and to analyse IOP changes according to different pathologies treated in the study.

## Methods

**Study design and setting:** a retrospective, observational, descriptive, uncontrolled single-centre series study was conducted at a tertiary centre in Lusaka, Zambia.

**Ethical considerations:** approval to conduct this study was granted by the Institutional Review Board (IRB) of Dr. Agarwal´s Eye Hospital in accordance with the Declaration of Helsinki. Informed written consent was obtained from patients to participate in the study.

**Study population:** patients who had undergone intravitreal DEX-I injection in one or both eyes from January 2016 to September 2018, with a minimum follow-up period of four months were included in the study. Exclusion criteria were known cases of steroid responder, pre-existing ocular hypertension, primary open angle glaucoma (POAG) patients, patients with family history of steroid response or glaucoma patients and indications for injection other than retinal vein occlusions, non-infectious posterior uveitis, DME and postsurgical macular edema.

**Data collection:** data entry included general demographics, ophthalmic and systemic history. Complete ocular examination at base line and at subsequent follow-up visits was done. Best corrected visual acuity (BCVA) was recorded in each visit on Snellen visual acuity chart. IOP was measured using Goldmann applanation tonometry, at baseline and at 1 week, 1^st^, 2^nd^, 3^rd^ and 4^th^ month after injection. Central macular thickness (CMT) in microns (μm) was measured after obtaining optical coherence tomography (OCT) image on Cirrus machine (Cirrus HD-OCT; Carl Zeiss Meditec, Dublin, CA) at each visit. Drugs used to treat secondary ocular hypertension and surgical intervention if any was noted.

**Definition:** ocular hypertension (OHT) was defined as IOP ≥ 21 mmHg or an increase of ≥ 10 mmHg over the follow-up period compared with baseline IOP.

**Data analysis:** data analysis was done to see the change in BCVA from baseline to 4^th^ month follow-up period, difference of IOP before and after treatment over the 4-month follow-up period, to measure the incidence of secondary OHT, to determine the difference in IOP as per the treatment indications and also the difference in CMT at baseline and 4^th^ month post injection. The efficacy of the DEX-I was assessed from gain in BCVA to > 20/60 Snellen (0.48 logMAR) and decrease in CMT to < 250μm or by >150μm from baseline macular thickness.

**Statistical analysis:** Snellen BCVA was converted to logMAR (logarithm of the minimum angle of resolution) decimal value for analysis. Qualitative (categorical) variables were described in terms of frequencies and percentages. Quantitative variables were described through the mean and standard deviation. Wilcoxon signed-rank test and Student´s t-test were used for comparing means. Univariate analysis was performed to determine the association between independent variable (age and sex) and IOP rise (dependent variable). The level of statistical significance was taken into account if p <0.05.

## Results

**Demographics:** the data pertains to 122 eyes of 99 patients, in 23 of which both eyes were treated with DEX-I. Mean age was 57.35 ± 13.87 years (range 16-80) and 65.65% patients were males (34 females).

**Injection and indications:** treatment indications were: DME in 52.45% (64 eyes), macular edema in retinal vein occlusion in 18.85% (23 eyes), post-surgical macular edema in 18.03% (22 eyes), macular edema in non-infectious posterior uveitis in 10.65% (13 eyes). Thirty-two-point seven percent (32.7%; 40 eyes) were pseudophakic at presentation and cataract surgery was done in 10.65% (13 eyes) during the course of the study. Mean follow-up duration was 9.13 ± 5.13 months (range 4 - 24 months). None of the eyes were retreated with DEX-I post 4^th^ month for persistent or recurrent edema. Rescue/re-treatment was done with anti-VEGF injections in 41 (33.60%) eyes (32 DME and 9 retinal vein occlusion). Systemic steroid for uveitis was started in 7 patients (minimum dose was 5 mg/day) during the follow-up period.

**Efficacy of implant:** the DEX-I used as primary indication for treatment of macular edema in this set of patients across all subtypes was efficacious. BCVA gained from a mean of 0.68 ± 0.47 logMAR at baseline to 0.33 ± 0.23 logMAR at 4^th^ month post-injection interval (p <0.0001; Wilcoxon signed - rank test). Corresponding decrease in mean CMT of 590.91 ± 202.53μm at baseline to a mean of 234 ± 41.14μm at 4^th^ month was achieved with the intravitreal implant (p <0.0001; Wilcoxon signed-rank test). Table 1 details the BCVA and CMT across subtypes of treated macular edema at baseline and 4^th^month with p-value.

**Table 1:** comparison of BCVA and CMT in different treated pathologies at baseline and 4^th^ month

Pathology treated	BCVA at baseline (logMAR)	BCVA at 4^th^ month (logMAR)	P - value	CMT at baseline (microns)	CMT at 4^th^ month (microns)	P-value
DME	0.73 ± 0.52	0.28 ± 0.27	<0.0001	474.6 ± 115.9	311.7 ± 41.83	0.04
RVO*	0.94 ± 0.58	0.43 ± 0.30	0.005	633.8 ± 235	297.4 ± 49.02	0.002
NIPU**	0.57 ± 0.37	0.31 ± 0.22	0.33	540.6 ± 198	217.7 ± 11.45	0.014
Post-surgical macular edema	0.78 ± 0.42	0.18 ± 0.28	0.001	598.2 ± 225.5	249.9 ± 38.66	0.003

* RVO: retinal vein occlusion; ** NIPU: non-infectious posterior uveitis

**Pressure response to implant:** the mean IOP before the injection was 14.73 ± 2.5 mmHg. Post-injection IOP was significantly different at 1 month, 2 months and 3 months (p<0.05; Wilcoxon signed-rank test) (Table 2). Overall, at all time intervals, the mean IOP was less than 20 mmHg.

**Table 2:** mean IOP at different time intervals post DEX-I

Intervals	IOP (mmHg)	P-value (Wilcoxon signed rank test)
Pre-op	14.73 ± 2.5	
1 week	14.45 ± 3.0	0.08
1 month	16.14 ± 4.6	0.01
2 months	17.36 ± 5.7	<0.0001
3 months	15.73 ± 3.7	0.006
4 months	14.92 ± 2.7	0.06

**Figure 1:**
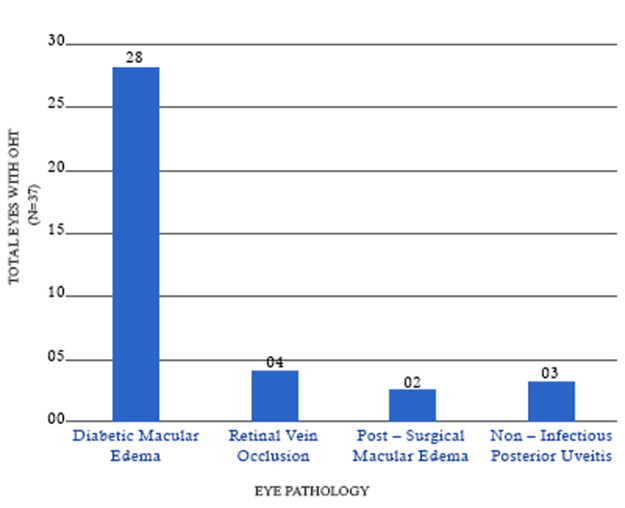
distribution of 37 eyes with ocular hypertension (OHT) across different treated pathology

**Incidence of ocular hypertension during follow-up:** OHT developed in 30.32% (37) eyes. The percentage of eyes with IOP >30 mm HG was 5.73% (n=7). Incidence of OHT was seen in 9.8% (12) eyes at 1 month, in 13.9% (17) at 2 months, in 4.09% (5) eyes at 3 month and in 2.45% (3) eyes at 4-month post-injection. Figure 1 demonstrates the distribution of eyes with OHT indication wise with majority, 75.67% being in the DME group (28 eyes). It also depicts, 43.75% of the eyes with DME, 23.07% eyes with non-infectious posterior uveitis, 17.39% eyes of retinal vein occlusion and 9.09% eyes with post-surgical macular edema developed OHT in this study. No significant association was found between age, gender and IOP change.

**Management of ocular hypertension:** all cases of OHT were managed with two anti-glaucoma medications (AGM)-FCBT (fixed combination Brimonidine/Timolol BD) and in 7 eyes with IOP >30 mmHg, a third AGM (Dorzolamide BD) was added. Ocular hypertension was transient and IOP returned to normal within 2 months of starting treatment. None of the eyes required systemic anti-glaucoma medications or filtration surgery.

## Discussion

This study evaluated the effect of DEX-I (Ozurdex) on a cohort of patients from Zambia in Southern Africa. To the best of our knowledge this is the first study to look at the IOP changes along with incidence of OHT following DEX-I in African eyes from a single centre comprising regional data as against recent studies which were multicentric or done on known glaucoma patients and or steroid responders [15-17].

Though the primary aim of the study was to evaluate the IOP response to DEX-I, the sub-analysis on outcome involving visual gain and central macular thickness decrease following a single dose of the implant showed its efficacy in treating macular edema in diabetes (DME), retinal vein occlusion, non-infectious posterior uveitis and post-surgery. DEX-I played a promising role in improving both visual acuity and macular anatomy in this Zambian cohort. DEX-I (Ozurdex) being a sustained delivery biodegradable intravitreal implant that releases corticosteroid for up to 6 months after intravitreal injection and one of the most potent corticosteroids (showing, anti-inflammatory activity sixfold higher than triamcinolone), Ozurdex may be recommended as a first choice for such cases in African eyes. However, as this study evaluated the outcome at short period of 4 months only, treating ophthalmologists should prepare the patients for repeat dosing in future for recurrence or persistence of macular edema.

The incidence of secondary ocular hypertension in this study was 30.3% which is comparable with SAFODEX^15^ (28.5%) and MEAD [13] study (approximately 33%), and higher than that found in GENEVA trial [12] (approximately 16%) and by Sudhalkar *et al*. [16] (23.3%). Significant IOP rise compared to baseline was noted at 2 months and as well the incidence of secondary ocular hypertension was highest at the same time with majority (29/37) of eyes having ocular hypertension from 1 month to 2-month post injection period. The maximum rise of IOP was noted within 60 days of implantation by the GENEVA study group [12], at 2 months post intravitreal injection in SAFODEX study [15], before 1.5-month to 2.5-month interval in the study by Chin *et al*. [14] and at 7 weeks reported by Lynds *et al*. [18]. The median time to onset of OHT was 48 days in the study by Sudhalkar *et al*. [16]. This study reflects the similar pattern of IOP rise. Besides the IOP trend found in present study where significant difference in IOP compared to baseline was noted up to 3^rd^ month only reiterates the same above observations. In fact 2.45% eyes had ocular hypertension at 4^th^ month post injection in the study. This delayed incidence highlights the fact that there can be a late peak in IOP as found in the study by Meyer *et al*. [19] which showed an onset of ocular hypertension as late as 8^th^month post injection. Our study had a mean follow-up period of 9 months and we didn´t observe any late steroid response following a single dose of DEX-I.

Although risk factors for ocular hypertension wasn´t assessed using regression analysis in this study, DME was the group with majority of cases with secondary ocular hypertension followed by non-infectious posterior uveitis. SAFODEX study [15] identified younger age, male sex, type 1 diabetes, pre-existing glaucoma treated with dual or triple therapy and eyes with retinal vein occlusion and non-infectious posterior uveitis as risk factors. The percentage of eyes with secondary ocular hypertension (43.75%) in the DME group was very high compared to the MEAD study [13] (27.7%) and SAFODEX study [15] (17%) and lesser than 63.2% incidence found by Srinivasan *et al*. [17] thus reflecting poor pressure tolerance profile in diabetic eyes treated with DEX-I. It is well known that ocular hypertension is a complication of uveitis and concomitant administration of topical and systemic steroids in NIPU could aggravate steroid-induced ocular hypertension [20]. Therefore, these two groups (DME and non-infectious posterior uveitis) need more close monitoring of IOP following DEX-I.

The secondary ocular hypertension was transient and well managed with topical anti-glaucoma medications resulting in IOP lowering within 2 months except for one case where IOP normalized by 6^th^ month. This is in accordance with past studies where IOP returned to normal within 6 months [5,12,13,15-17,21]. In this study all patients with secondary ocular hypertension were started straight away on 2 anti-glaucoma medications and 3 anti-glaucoma medications were used in 18.91% patients (7/37) as against in study by Sudhalkar *et al*. [16] where 31% needed 2 drugs, 10% needed triple therapy; 31% of eyes needed IOP-lowering medications in SAFODEX study [15] and 78.6% cases were managed with one anti-glaucoma medication only in study by Srinivasan *et al*. [17]. This reflects lower threshold exercised on our part to manage steroid induced ocular hypertension with glaucoma monotherapy only.

None of the eyes required filtration surgery. This implies better response of ocular hypertension to medications in this group compared to data from other studies where trabeculectomy surgery was needed in 0.7-1% cases [1,3,15]. This study again highlights an overall good pressure tolerance of DEX-I compared to very high incidence of ocular hypertension with other intravitreal steroidal drugs like triamcinolone acetonide 4mg (40% OHT) and fluocinolone acetonide 190mg (38% ocular hypertension with high responders 18%) [22,23]. This may be attributed to different pharmacologic and pharmacokinetic profiles of intravitreal steroids with regard to ocular hypertension. Dexamethasone, fluocinolone acetonide and triamcinolone have been shown to activate different patterns of gene expression in human trabecular meshwork cell lines [24].

Dexamethasone differs from triamcinolone in pharmacologic activity, lipid solubility, and delivery requirements. Dexamethasone is less lipophilic and does not accumulate to the same extent in the trabecular meshwork and therefore may have lower risk of IOP increases [25,26]. Notable limitations of this study were the small sample size and retrospective design. The study didn´t assess the risk factors of ocular hypertension following DEX-I in this cohort. Rescue or retreatment with DEX-I injection cases were not included and thus cumulative effect on IOP in “multi-injected eyes” could not be evaluated. Association of ocular hypertension and position of DEX-I in vitreous cavity should have been studied.

## Conclusion

Nevertheless, our data showed that DEX-I is not only effective in treating macular edema with good functional and anatomical outcome but also has a safe profile in terms of IOP in this regional cohort. The peak IOP change occurs at 2 months following injection. Nearly 1/3^rd^ eyes are expected to develop secondary ocular hypertension, majority of them by 2 months, which can be well managed with medications alone. Intraocular pressure normalises within 6 months following intravitreal dexamethasone implant. Eyes with DME and non-infectious posterior uveitis are more susceptible to develop secondary ocular hypertension, therefore close follow-up is warranted in such cases in the post-operative period. However, a study with larger sample size is needed to conclusively establish the IOP characteristics of intravitreal dexamethasone implant in various pathologies.

### What is known about this topic

- Very few studies across the world have shown incidence of secondary ocular hypertension following DEX-I injection intravitreally is 16-33%;
- Maximum IOP rise following injection is observed within 60 days;
- Only one study has evaluated risk factors for secondary ocular hypertension with type 1 DME, retinal vein occlusion and non-infectious posterior uveitis as the major ones.

### What this study adds

- This is the first study to assess the efficacy and safety of dexamethasone implant in terms of IOP in an African cohort;
- Eyes with diabetic macular edema (DME) and macular edema secondary to uveitis may have higher IOP rise following DEX-I;
- Pressure tolerance of DEX-I in this cohort (Zambian eyes) was good.
